# Granular Cell Tumor over the Mons Pubis: An Uncommon Tumor

**DOI:** 10.1155/2019/1279137

**Published:** 2019-05-15

**Authors:** M. Patabendige, D. J. Wickramasooriya, L. Dasanayake

**Affiliations:** ^1^University Unit of Obstetrics and Gynaecology, Teaching Hospital, Mahamodara, Galle, Sri Lanka; ^2^Department of Pathology, Faculty of Medicine, University of Ruhuna, Galle, Sri Lanka; ^3^Department of Obstetrics and Gynaecology, Faculty of Medicine, University of Ruhuna, Galle, Sri Lanka

## Abstract

Granular cell tumors are uncommon, usually benign, soft tissue neoplasms of neural origin. They occur throughout the body; vulval involvement is uncommon and labium majus is the commonest site in vulva. Complete surgical excision is the preferred treatment of choice to prevent recurrence. Here, we present a benign granular cell tumor over the mons pubis of vulva in a 27-year-old woman.

## 1. Introduction

Granular cell tumors (GCT) are uncommon slow-growing soft tissue tumors, considered to be derived from peripheral nerves, particularly Schwann cells [1]. These are often asymptomatic [1]. Most cases are sporadic and are located in the subcutaneous layer [2]. Macroscopically, GCT are small, firm, solitary whitish nodules with poor encapsulation [2]. Vulvar involvement is uncommon [3]. Commonest reported site of GCT in the female genital tract is on the labium majus [2]. Although most vulval GCT are benign, about 1 to 2% of the cases are malignant and may be associated with regional or distant metastases [4, 5]. The aggressive malignant form of GCT exhibits a poor response to radiotherapy and chemotherapy [6]. This case describes a benign GCT over the mons pubis of vulva in a 27-year-old woman. This location of mons pubis is hardly reported in the literature and only two cases were found in English literature [3, 7].

## 2. Case Report

A 27-year-old nulliparous woman presented with an increasing vulval lump for 2-year duration without any significant associated symptoms. She has no history of pain, discharge, or any bleeding from the area. She denied any history of previous vulvar lesion or any other systemic symptoms. Her past history and family history revealed nothing significant.

**Figure 1:**
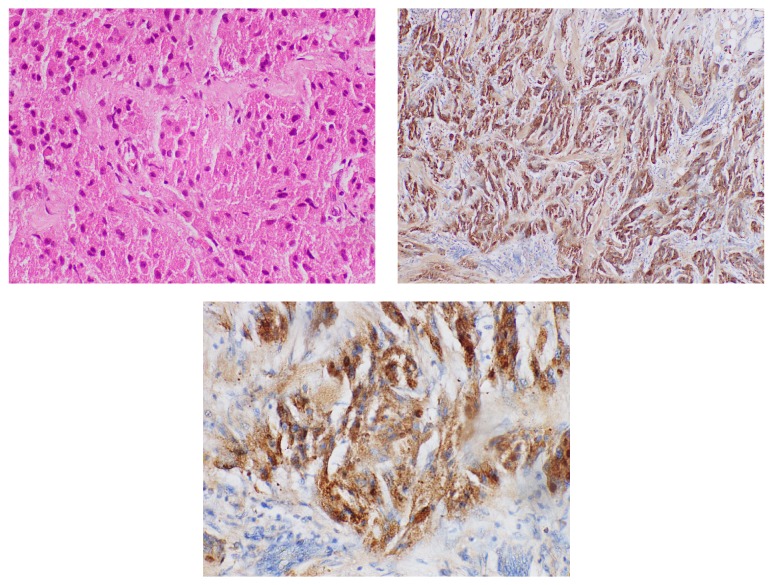
Tumor cells with large and polygonal with abundant eosinophilic granular cytoplasm and small nuclei. (a) Haematoxylin and Eosin staining. (b) and (c) Immunohistochemical staining (S-100), respectively.

The physical examination revealed a hard, mobile, nodular, subdermal, mass measuring 30 × 20 mm over the mons pubis and it was about 2 cm superior to the clitoris and 1 cm lateral to the midline. It was fixed to the overlying skin which had a normal appearance. The lesion was nontender and on palpation there was no sign of discharge or bleeding. There were no palpable lymph nodes. Preoperative ultrasound scan showed no increased vascularity around the lump. An excision biopsy of the lesion was performed under regional anaesthesia. Macroscopically, the specimen measured 30 × 20 mm. The overlying skin was unremarkable. On slicing, the cut surface was pale tan. Microscopy revealed a poorly circumscribed, unencapsulated tumor composed of nests and strands of polygonal cells having abundant eosinophilic granular cytoplasm and small, central hyperchromatic nuclei. Cell margins are indistinct. The margins appear infiltrative. However, there is no nuclear or cytological atypia. There are no mitoses and excision margins are free of tumour. Immunohistochemistry showed that the tumor cells are S-100 positive. These features were compatible with a GCT without features of malignancy. On the follow-up appointment at 4 months after the excision procedure, the patient was asymptomatic. Microscopic appearance with Haematoxylin and Eosin and immunohistochemical staining has been shown in Figure 1.

## 3. Discussion

Even though the most common location of GCT in the female genital tract is on the labium majus, it was over the mons pubis in this case. Microscopically, GCT are composed of loosely infiltrating sheets or clusters of large round or polygonal spindled cells with abundant eosinophilic cytoplasm with intracytoplasmic granules as shown in Figure 1 [1]. The resection margins should be adequately taken for a proper treatment and follow-up. Recurrence is more likely in a tumour with infiltrated margins [2].

The differential diagnoses include other granular cell variants such as basal cell carcinoma, melanoma, leiomyoma, leiomyosarcoma, dermatofibrosarcoma, angiosarcoma, fibrous histiocytoma fibroma, lipoma, and hidradenoma [1]. Moreover, histologically GCT may be difficult to distinguish from other granular cell variants of various tumors if examined with routine light microscopy alone. Immunohistochemistry aids in distinguishing GCT from other similar types. GCT stains negative for desmin, cytokeratins, epithelial membrane antigen, and glial fibrillary acidic protein [1]. Immunohistochemical stains in GCT are positive for S-100 protein, CD68, periodic acid Schiff (PAS), neuron-specific enolase, peripheral nerve myelin proteins, and vimentin and are diastase resistant [8–10]. Therefore, it helps to distinguish it from other differential diagnoses. Since the positivity for S-100 protein and neuron-specific enolase is high, currently it is thought that this tumor originates from Schwann cells. However, Schwannomas show biphasic histopathological appearance: compact hypercellular Antoni A areas and myxoid hypocellular Antoni B areas [11]. Also, nuclear palisading around fibrillary process (Verocay bodies) is often seen in cellular areas. In contrast, GCT lack these features and their large polygonal cells contain a highly granular cytoplasm occupying the entire lesion [5]. Basically, these help to differentiate Schwannoma from a GCT microscopically.

Nuclear hyperchromatism and pleomorphism and engorgement of the cytoplasm with complex granules are elements of the malignant variant of this tumor [1, 9]. Our case also had some of these features. In histological point of view, the specific criteria for malignancy have been described. These are necrosis, spindling, vesicular nuclei with increased mitotic activity, high nuclear to cytoplasmic ratio, and nuclear pleomorphism [12, 13]. GCT that meet at least three of these criteria can be considered as histologically malignant [12, 13]. Malignant GCTs are often immunohistochemically negative for S-100 protein, neuron-specific enolase, and vimentin [9]. Therefore, our case was labeled as benign. However, the distinction between benign and malignant GCT is difficult because of histologic similarity and lack of reliable criteria that can predict clinical behavior [1]. We had this dilemma while interpreting the slides.

Surgery remains the primary treatment modality for GCT. Local surgical excision is generally curative for benign tumours. Wider local excision is recommended to reduce the risk of recurrence. In cases with resection, margins are found to be positive [2]. In our case, surgical specimen had negative margins. However, our patient was scheduled for six-month follow-up for any local recurrence or extragenital recurrence.

In summary, this case describes a GCT over the mons pubis in female genital tract and this location is hardly reported in the literature. However, these uncommon and mostly benign vulval tumours have a tendency for recurrence. During follow-up, other areas such as oral cavity and trunk also should be carefully examined. Once diagnosed with a GCT, the patient has to be counseled for regular follow-up. They should be advised to seek early medical attention if any growth recurs at the excision site or if any nodular growth develops elsewhere on the body.

## References

[1] Hong S., Lim Y., Chew S., Chia Y., Yam K. (2013). Case report of granular cell tumor of the vulva and review of current literature. *Gynecologic Oncology Reports*.

[2] Althausen A. M., Kowalski D. P., Ludwig M. E., Curry S. L., Greene J. F. (2000). Granular cell tumors: a new clinically important histologic finding. *Gynecologic Oncology*.

[3] Clark N., Thieu T., Zuckerman A., Jacobsen L. J. (2014). Benign granular cell tumor of the mons pubis in an 8-year-old girl. *Journal of Pediatric & Adolescent Gynecology*.

[4] Simone J., Schneider G. T., Begneaud W., Harms K. (1996). Granular cell tumor of the vulva: literature review and case report. *Journal of the Louisiana State Medical Society*.

[5] Rose B., Tamvakopoulos G. S., Yeung E. (2009). Granular cell tumours: A rare entity in the musculoskeletal system. *Sarcoma*.

[6] Schmidt O., Fleckenstein G. H., Gunawan B., Füzesi L., Emons G. (2003). Recurrence and rapid metastasis formation of a granular cell tumor of the vulva. *European Journal of Obstetrics & Gynecology and Reproductive Biology*.

[7] Aniebue U., Olusina B. (2009). Granular cell tumor - a rare tumor of the mons pubis: case report and literature review. *Tropical Journal of Obstetrics and Gynaecology*.

[8] Levavi H., Sabah G., Kaplan B., Tytiun Y., Braslavsky D., Gutman H. (2006). Granular cell tumor of the vulva: Six new cases. *Archives of Gynecology and Obstetrics*.

[9] Mosbeh S., Shaaban D. (2012). Vulval granular cell tumor: a rare entity. *The Gulf Journal of Dermatology and Venereology*.

[10] Goldblum Lamps L., McKenney J., Myers J. (2017). *Rosai and Ackerman's Surgical Pathology*.

[11] Rodriguez F. J., Folpe A. L., Giannini C. (2012). Pathology of peripheral nerve sheath tumors: diagnostic overview and update on selected diagnostic problems. *Acta Neuropathologica*.

[12] Fanburg-Smith J. C., Meis-Kindblom J. M., Fante R., Kindblom L.-G. (1998). Malignant granular cell tumor of soft tissue: diagnostic criteria and clinicopathologic correlation. *The American Journal of Surgical Pathology*.

[13] Kim H. J., Lee M.-G. (2015). Granular cell tumors on unusual anatomic locations. *Yonsei Medical Journal*.

